# Does psychological distress influence postoperative satisfaction and outcomes in patients undergoing total knee arthroplasty? A prospective cohort study

**DOI:** 10.1186/s12891-021-04528-7

**Published:** 2021-07-30

**Authors:** Tao Bian, Hongyi Shao, Yixin Zhou, Yong Huang, Yang Song

**Affiliations:** Department of Orthopedic Surgery, Beijing Jishuitan Hospital, Fourth Clinical College of Peking University, No. 31 Xinjiekou East Street, Xicheng District, Beijing, 100035 China

**Keywords:** Psychological factor, Postoperative outcome, Patient satisfaction, Total knee arthroplasty, Psychological distress

## Abstract

**Background:**

Preoperative psychological distress may be related to dissatisfaction and poorer outcomes after total knee arthroplasty (TKA). However, the kind of psychological distress that could influence postoperative satisfaction and outcomes remains controversial. Few studies have examined these issues in Chinese cohorts. Thus, this study aimed to examine (1) the prevalence of preoperative psychological distress in patients undergoing TKA and (2) whether preoperative psychological distress influences patient satisfaction, early postoperative outcomes, and improvement of knee function after TKA.

**Methods:**

We prospectively included 210 patients undergoing unilateral primary TKA between March 2017 and September 2017 at our institution. Preoperatively, patients completed the Depression Anxiety and Stress Scales and new Knee Society Scores (KSS) questionnaires. At 3 months and 1 year postoperatively, patients’ KSS and overall satisfaction were assessed. Stepwise multivariate linear regression models were used to assess the variables that influenced changes in each KSS item.

**Results:**

Preoperatively, 89 (42.4%) patients experienced psychological distress. The satisfaction rate and postoperative KSS were not significantly different between patients with or without psychological distress; a higher preoperative score was shown to predict less KSS improvement. Patients with depression had fewer symptom score changes.

**Conclusions:**

The prevalence of preoperative psychological distress was relatively high; thus, surgeons should consider the patient’s psychological state. Patients’ satisfaction was not influenced by psychological factors. Patients with depression and higher preoperative scores had lower symptom scores and KSS improvement, respectively.

**Supplementary Information:**

The online version contains supplementary material available at 10.1186/s12891-021-04528-7.

## Background

Total knee arthroplasty (TKA) can effectively treat end-stage knee arthritis. In the United States, more than 3.4 million TKAs will be performed annually by 2030 [1]. However, a study showed that approximately 20% of the patients are dissatisfied with their postoperative outcomes [2]. Many factors can influence patient satisfaction, including patient expectations, preoperative knee function, and pain [2]. Several recent studies have reported that a patient’s preoperative psychological distress is associated with dissatisfaction and poorer patient-reported outcome measures (PROMs) [3–6].

However, some previous studies have reported a relatively low response rate to mental problems, which may have affected their results [3–5]. Others have used general mental health measurements instead of specific psychological distress parameters, such as depression, anxiety, or stress, to investigate the influence of psychological distress on the outcome and satisfaction of patients undergoing TKA [3, 4]. Moreover, such studies focusing on Chinese patients are limited, and it is noteworthy that different ethnicities may have different psychological problems owing to varying cultural backgrounds.

Thus, the aims of this study were to examine (1) the prevalence of preoperative psychological distress in patients undergoing TKA in our institution and to determine (2) whether preoperative psychological distress influences patient satisfaction, early postoperative outcomes, and improvement of knee function following TKA.

We hypothesized that the prevalence of preoperative psychological distress is relatively high in patients undergoing TKA, and preoperative psychological distress influences patient satisfaction, postoperative outcomes, as well as knee function improvement.

## Methods

A single-center, prospective cohort study was conducted in our institution between March 2017 and September 2017, wherein patients undergoing primary unilateral TKA were consecutively evaluated for enrollment. The study was approved by the Institutional Review Board of our hospital, and all patients included in this study provided informed consent prior to recruitment. The inclusion criteria were as follows: aged ≥ 18 years and diagnosed with osteoarthritis. The exclusion criteria were as follows: revision operation, bilateral TKA, previous knee infection or implant operation on the same side, and inability to complete the questionnaires because of cognitive or language difficulties.

Demographic data including sex, age, weight, height, presence or absence of family support, residency status in Beijing, waiting time for the operation, Charlson comorbidity index [7, 8], and use of any medication were collected preoperatively. Body mass index (BMI) was calculated using the patients’ weight and height. Patients’ mental health and knee conditions were evaluated before the operation using the Depression Anxiety and Stress Scales (DASS) 21 and the new Knee Society Scores (KSS), respectively. Patients’ knee conditions and overall satisfaction with the surgical outcome were evaluated using the new KSS and 5-point Likert scale at 3 months and 1 year postoperatively, respectively. Patients were asked to answer the evaluation forms, and in cases where the patients were not able to visit the hospital, we interviewed them via telephone and filled out their forms.

The DASS is a validated questionnaire that evaluates the psychological traits of depression, anxiety, and stress [9]. In our study, we used the Short Form-21 version (Online Resource 1). Patients answered each statement and rated their responses from 0 (does not apply to me at all) to 3 (applied to me very much) points. Each subscale (depression, anxiety, and stress) had seven questions, and we summed up the scores separately and then multiplied the value by two according to the scale instructions. The questionnaire has its recommended cut-off score for each subscale, which is 9 points for depression, 7 points for anxiety, and 14 points for stress. Using the aforementioned cut-off scores, we dichotomized the patients to determine whether they were depressed, anxious, or stressed out prior to the surgery. In our study, the patients were divided into the following two groups according to their mental health status: the distressed group included patients with either depression, anxiety, or stress (in accordance with a previous study’s definition of distressed patients [10]) and the non-distressed group comprised patients without the aforementioned psychological problems. Furthermore, we subdivided patients in the distressed group into different categories based on severity (mild, moderate, severe, extremely severe) according to the cut-off values in the questionnaire’s instructions (Online Resource 1). The simplified Chinese version of the DASS is validated and has a high internal consistency [11].

The new KSS was developed in 2012, and several studies have confirmed its reliability, validity, and internal consistency [12–14]. In our study, we used the simplified Chinese version of the KSS, which was translated by Liu et al. [14]. It consists of the following parts: objective knee score, satisfaction score, expectation score, and functional activity score [15]. We further divided objective knee score into two parts: objectives and symptoms, based on whether the form was completed by the doctor or the patient, following which these were included in the analysis. For every part of the KSS, higher scores indicated better outcomes.

Patient overall satisfaction was measured using a 5-point Likert scale. During the analysis, we divided the results into two categories: satisfied or dissatisfied. The answers “very satisfied” and “satisfied” indicated satisfaction, whereas “neutral,” “dissatisfied,” and “very dissatisfied” were considered dissatisfaction.

### Statistical analysis

Demographic data were compared between the distressed and non-distressed patients. Pearson’s chi-square test or Fisher’s exact test was used to compare categorical variables. For continuous variables, the Shapiro–Wilk test was used to determine whether variables were normally distributed, whereas independent Student’s t-test or Mann–Whitney U test was used depending on the normality of the variables.

The Kruskal–Wallis H test was used to compare each part of the KSS in patients with different distress characteristics and severity categories at different time points, as the variables did not have a normal distribution. Post hoc pairwise comparisons were used to further identify significant group differences. The Wilcoxon signed rank test was used to assess changes in each part of KSS before and after the surgery. Then, stepwise multivariate linear regression models were used to assess which variables influence the changes in KSS. The independent variables were demographic data, five parts of preoperative KSS, and severity categories of depression, anxiety, and stress. Dependent variables were the score changes of each part of the KSS between 1-year postoperatively and preoperatively.

All statistical analyses were performed using SPSS 20.0 (SPSS Inc., Chicago, IL, USA). For all comparisons and regressions, significance was set at *P* < 0.05.

## Results

**Fig. 1:**
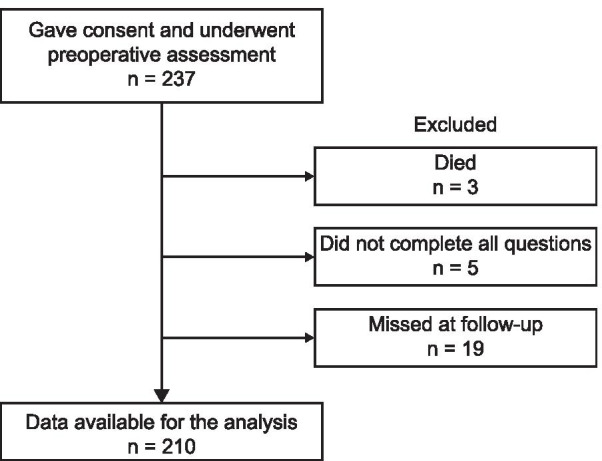
Flowchart showing the study cohort and those patients excluded from the analysis

Altogether, 237 patients consented to participate in this study. At the 1-year follow-up evaluation, 3 patients died due to reasons unrelated to the joint procedure, 5 patients did not answer all questions, and 19 patients were lost during follow-up. Finally, 210 patients who were successfully followed up were included in the final analysis (Fig. 1). The mean age was 65.7 ± 7.9 (range, 43–85) years. There were 28 (13.3%) men and 182 (86.7%) women. The median BMI was 26.4 (interquartile range [IQR], 24.1–28.9) kg/m^2^. The median waiting days for operation was 10.0 (IQR, 5.8–20.0) days. No patient was diagnosed with a psychological disorder or was on medication for chronic depression or anxiety prior to the operation. There were more women in the distressed group (*P* = 0.046). The demographic data are listed in Table 1.

**Table 1:** Patient demographics (n = 210)

Measure	Distressed group n = 89	Non-distressed group n = 121	*P* value
Mean age, years (range)	64.7 ± 7.1 (47.0 to 79.0)	66.5 ± 8.4 (43.0 to 85.0)	0.111
Sex, n (%)			
Female	82 (92.1%)	100 (82.6%)	0.046
Male	7 (7.9%)	21 (17.4%)	
Median body mass index, kg/m^2^ (IQR)	27.2 (24.0 to 29.1)	26.1 (24.4 to 28.6)	0.328
Had family support, n (%)	84 (94.4%)	113 (93.4%)	0.768
Living in Beijing, n (%)	40 (44.9%)	51 (42.1%)	0.686
Median waiting days for operation, (IQR)	9.0 (6.0 to 18.5)	10.0 (5.0 to 22.0)	0.402
Charlson index, n (%)			
0	0	2 (1.7%)	0.459
1	17 (19.1%)	16 (13.2%)	
2	32 (36.0%)	47 (38.8%)	
3	29 (32.6%)	38 (31.4%)	
4	8 (9.0%)	15 (12.4%)	
5	1 (1.1%)	3 (2.5%)	
6	2 (2.2%)	0	

*Abbreviation*: *IQR* interquartile range

### Prevalence of psychological distress

Among the 210 patients included in the analysis, 89 (42.4%) patients experienced at least one psychological distress parameter, whereas 121 (57.6%) patients did not experience any psychological distress. The distribution of patients in different types and severity categories of psychological distress are shown in Table 2.

**Table 2:** Patient distributions in different types and severity categories of psychological traits

	Extremely severe	Severe	Moderate	Mild	Normal
Depression n (%)	0	3 (1.4%)	13 (6.2%)	23 (11.0%)	171 (81.4%)
Anxiety n (%)	10 (4.8%)	15 (7.1%)	45 (21.4%)	16 (7.6%)	124 (59.0%)
Stress n (%)	0	4 (1.9%)	6 (2.9%)	16 (7.6%)	184 (87.6%)

### Relationship between preoperative psychological distress and postoperative patient satisfaction

No statistical difference was found between patients not facing distress and those facing different numbers of distress characteristics in terms of the dissatisfaction rate at 1 year (*P* = 0.760) postoperatively. No difference was observed in the dissatisfaction rate in the separate analyses for different types and severity categories of psychological traits (Table 3).

**Table 3:** Distress characteristics and types and severities of psychological traits in patients stratified by postoperative satisfaction

	Dissatisfied n = 33 (15.7%)	Satisfied n = 177 (84.3%)	*P* value
One year postoperatively, n (%)			
Three distress characteristics, n = 18	4 (22.2%)	14 (77.8%)	0.760
Two distress characteristics, n = 26	4 (15.4%)	22 (84.6%)	
One distress characteristic, n = 45	8 (17.8%)	37 (82.2%)	
No distress, n = 121	17 (14.0%)	104 (86.0%)	
Severe depression, n = 3	1 (33.3%)	2 (66.7%)	0.115
Moderate depression, n = 13	1 (7.7%)	12 (92.3%)	
Mild depression, n = 23	7 (30.4%)	16 (69.6%)	
No depression, n = 171	24 (14.0%)	147 (86.0%)	
Extremely severe anxiety, n = 10	1 (10.0%)	9 (90.0%)	0.894
Severe anxiety, n = 15	2 (13.3%)	13 (86.7%)	
Moderate anxiety, n = 45	9 (20.0%)	36 (80.0%)	
Mild anxiety, n = 16	3 (18.8%)	13 (81.3%)	
No anxiety, n = 124	18 (14.5%)	106 (85.5%)	
Severe stress, n = 4	1 (25.0%)	3 (75.0%)	0.646
Moderate stress, n = 6	0 (0.0)	6 (100.0%)	
Mild stress, n = 16	3 (18.8%)	13 (81.3%)	
No stress, n = 184	29 (15.8%)	155 (84.2%)	

### Relationship between psychological distress and KSS

Patients had a significant improvement in the median function (34.0 preoperatively vs. 55.0 postoperatively), symptom (8.0 preoperatively vs. 25.0 postoperatively), objective (27.0 preoperatively vs. 65.0 postoperatively), and satisfaction (14.0 preoperatively vs. 30.0 postoperatively) scores at 1 year postoperatively (all *P* < 0.001). Among patients facing different distress characteristics and those without distress, a difference was found in the preoperative function score and change of function score when performing the Kruskal–Wallis H tests. However, no significant difference was observed in the pairwise comparisons (Online Resource 2).

A difference was noted in the preoperative satisfaction score among patients with or without depression and patients with or without anxiety when performing the Kruskal–Wallis H tests. However, no significant difference was found in the pairwise comparisons. No significant difference was also found in the other analyses among patients with different types and severity categories of psychological distress (Online Resources 3, 4 and 5).

When using the multivariate linear regression model to predict the change in the KSS, different variables were included in each final model. For the Knee Society function score, the change in score depended on the preoperative Knee Society function score (R^2^ = 0.703; β = -0.965; *P* < 0.001) alone, whereas the change in the Knee Society symptom score (R^2^ = 0.799) depended on the preoperative Knee Society symptom score (β = -1.063; *P* < 0.001) and the severity of depression (β = -0.928; *P* = 0.002). Patients with more severe depression would have a reduced increase in Knee Society symptom score. The change in Knee Society objective score (R^2^ = 0.723) depended on the preoperative Knee Society objective score (β = -0.912; *P* < 0.001) and sex (β = -6.933; *P* = 0.008). Men had a reduced increase in Knee Society objective score. For all regression models, higher preoperative score could predict fewer increases in the same part of the Knee Society score (Table 4).

**Table 4:** Linear regression of variables with significant values

Dependent variables	Predictor variables	Beta coefficient	*P* value
Changes in Knee Society function score	Preoperative Knee Society function score	-0.965	< 0.001
Changes in Knee Society symptom score	Preoperative Knee Society symptom score	-1.063	< 0.001
	Severity of depression	-0.928	0.002
Changes in Knee Society objective score	Preoperative Knee Society objective score	-0.912	< 0.001
	Sex	-6.933	0.008

## Discussion

In this study, approximately 40% of patients who underwent TKA experienced psychological distress. However, it was not related to postoperative dissatisfaction. We found that preoperative depression influenced the improvement of Knee Society symptom score.

In our study, 42% of the patients experienced psychological distress; this incidence rate was higher than that reported in previous studies [10, 16–18]. The difference may be attributed to ethnic differences. In China, the prevalence of depressive disorders is reportedly 6.8% and that of an anxiety disorder is 7.6% [19]. In Chinese patients with severe osteoarthritis scheduled for TKA, Geng et al. reported that the prevalence of depression was 8.83% [20]. However, mental disorders were diagnosed by psychiatrists after a clinical interview in the abovementioned two studies. In the present study, we used a patient self-reported questionnaire to screen for psychological distress, which was a less strict criterion. Furthermore, we found that most patients were anxious before the surgery. Anxiety is the state of feeling nervous or worried that something ill is going to happen. This may explain why most of the patients were anxious, as it is natural to be worried about an upcoming surgical procedure. Furthermore, Fehring et al. reported that patients waiting for TKA may experience situational depression, which was largely caused by chronic pain and physical disability due to advanced arthritis [21]. The reasons above may lead to the higher prevalence of psychological distress in our study. It is worth noting that no patient in our study reported a previously diagnosed psychological disorder or was on a psychiatric drug. This may be due to the low awareness rates, diagnostic rates, and treatment rates of mental disorders in Chinese patients [22]. Additionally, we found that a higher proportion of women were present in the distressed group than in the non-distressed group, which was similar to that in previous studies [23, 24]. However, the proportion of women was relatively high in our study; the reason for that remains unknown and further investigation may be needed.

Khatib et al. conducted a systematic review and found that psychological health was a predictor of satisfaction after TKA in four studies [3–5, 25, 26], which reported that patients with psychological distress were more likely to be dissatisfied with the results. Besides, Ali et al. reported that patients with psychological distress had a > 6 times higher risk of being dissatisfied [6]. In contrast to previous studies, we found that the patient satisfaction rate was similar among different groups. Previous studies have reported a relatively low response rate, which may have caused a response bias and influenced the final results. For our study, the response rate was nearly 90%, which could have minimized the response bias. Furthermore, previous studies have used the European Quality of Life-5 Dimensions, mental component score of Short Form-36, or Short Form-12 to evaluate psychological distress. These questionnaires are focused on patients’ quality of life and their general mental health state, but not on the specific psychological problems. We used the DASS 21 to evaluate depression, anxiety, and stress, which more precisely reflected the patients’ psychological state. However, we did not find a difference in either combined analysis or separated analysis. Besides, the aforementioned studies have included Caucasian patients, and our study included Asian patients, which may also account for the differences.

Some studies have reported that poorer preoperative emotional health was associated with less improvement in physical function after TKA [25, 27]. By contrast, several studies have found that preoperative psychological distress did not influence the outcome and degree of improvement after operation [23, 28–31]. In our study, patients with psychological distress and those without distress had a similar degree of improvement. Besides, in the multivariate linear regression, the difference in Knee Society function score and objective score was not influenced by preoperative psychological factors. Knee Society function, symptom, and objective scores were significantly influenced by the preoperative score, with higher preoperative score resulting in lesser increases. The results of our linear regression analysis were similar to those of a study conducted by Utrillas-Compaired et al. [16]. However, they also reported that preoperative depression influences the improvement in knee function, which was inconsistent with our findings. We found that patients with preoperative depression exhibited less improvement in the Knee Society symptom score. Given that the Knee Society symptom score is more focused on patients’ subjective feelings and pain perception may be related to psychological status, this may be the reason why depression could cause such a difference. Thus, more studies related to this topic are needed to validate this finding.

For the three linear models reported, the R^2^ values ranged from 0.703 to 0.799, indicating that the included variables could explain 70.3% to 79.9% of the improvement in each part of the KSS. These values are relatively high.

This study has several limitations. First, this was a single-institution study and included Chinese patients alone, which may limit the generalizability of the results. Additionally, the result of a 4% difference in the dissatisfaction rate between patients with or without distress was obtained from an analysis using a limited sample size. Owing to the different cultural backgrounds, these results may be quite different from those of studies conducted in Western countries. However, to our knowledge, this was a prospective study focusing on Chinese patients with a relatively large sample size. Given that the DASS 21 and new KSS have been validated in Chinese patients, data in this study are comparable with those in previous studies. Second, we only followed up patients for 1 year; TKA outcomes may change further after 1 year. However, previous studies have reported that the level of satisfaction and PROMs are stable at 1 year after surgery, indicating that the 1-year follow-up duration was sufficient [28, 32]. Third, we used a patient self-reported questionnaire to evaluate the patients’ psychological state rather than a formal clinical assessment conducted by a psychiatrist, which was relatively superficial. Moreover, we only evaluated three types of psychological distress. However, our method allowed the clarification of the patients’ psychological state, and a formal clinical assessment is quite time consuming. Most previous studies have utilized self-reported questionnaires to screen for psychological distress [3–5]. We performed the DASS 21 preoperatively rather than in advance of the TKA surgical diagnosis, and we did not investigate other exogenous events, preoperative non-operative treatment methods, and postoperative psychological states. Recent studies have reported that patients’ psychological states may improve after TKA [17, 21, 33–36]. We focused more on the preoperative psychological state and its relationship with the outcome rather than the improvement in the psychological state.

## Conclusions

We found that approximately 40% of patients who underwent TKA experienced psychological distress. At 1-year post-TKA, approximately 15% of the patients were dissatisfied of the outcome, which was not influenced by psychological factors. Moreover, the improvement of each part of the KSS was influenced by the preoperative score. Depression was associated with lower postoperative symptom score improvement. Surgeons should consider the patient’s psychological state, as psychological distress is highly prevalent in patients undergoing TKA.

## Supplementary Information

**Additional file 1: Online Resource 1.** Depression Anxiety and Stress Scales 21 questionnaire.

**Additional file 2: Online Resource 2. **Knee Society Scores at different time points among patients with different numbers of distress characteristics.

**Additional file 3: Online Resource 3. **Knee Society Scores at different time points among patients with different severity scores of depression.

**Additional file 4: Online Resource 4. **Knee Society Scores at different time points among patients with different severity scores of anxiety.

**Additional file 5: Online Resource 5. **Knee Society Scores at different time points among patients with different severity scores of stress.

## Data Availability

The datasets used and/or analyzed during the current study are available from the corresponding author on reasonable request.

## Abbreviations

BMI: Body mass index
DASS: Depression anxiety and stress scales
KSS: Knee Society Score
PROM: Patient-reported outcome measures
TKA: Total knee arthroplasty
