# Supplementary material for: Does psychological distress influence postoperative satisfaction and outcomes in patients undergoing total knee arthroplasty? A prospective cohort study
Source: BMC Musculoskelet Disord. 2021 Jul 30;22:647. doi: 10.1186/s12891-021-04528-7 (PMC8325222; doi:10.1186/s12891-021-04528-7)
Supplement: Supplementary file 1 — Additional file 1: Online Resource 1. Depression Anxiety and Stress Scales 21 questionnaire. [file 12891_2021_4528_MOESM1_ESM.pdf]

Article title: Does psychological distress influence postoperative satisfaction and outcomes in patients undergoing total knee arthroplasty? A prospective cohort study

Journal name: BMC Musculoskeletal Disorders

Author names: Tao Bian, Hongyi Shao, Yixin Zhou, Yong Huang, Yang Song

Corresponding Author: Yixin Zhou
Department of Orthopedic Surgery, Beijing Jishuitan Hospital, Fourth Clinical College of Peking University, No. 31 Xinjiekou East Street, Xicheng District, Beijing 100035, China
E-mail: orthoyixin@yahoo.com

Online Resource 1. The Depression Anxiety and Stress Scales 21 questionnaire

DASS 21

Please read each statement and circle a number 0, 1, 2 or 3 which indicates how much the statement applied to you *over the past week*. There are no right or wrong answers. Do not spend too much time on any statement.

The rating scale is as follows:

0 Did not apply to me at all
1 Applied to me to some degree, or some of the time
2 Applied to me to a considerable degree, or a good part of time
3 Applied to me very much, or most of the time

						subscale
1	I found it hard to wind down	0	1	2	3	S
2	I was aware of dryness of my mouth	0	1	2	3	A
3	I could not seem to experience any positive feeling at all	0	1	2	3	D
4	I experienced breathing difficulty (e.g., excessively rapid breathing, breathlessness in the absence of physical exertion)	0	1	2	3	A
5	I found it difficult to work up the initiative to do things	0	1	2	3	D
6	I tended to over-react to situations	0	1	2	3	S
7	I experienced trembling (e.g., in the hands)	0	1	2	3	A
8	I felt that I was using a lot of nervous energy	0	1	2	3	S
9	I was worried about situations in which I might panic and make a fool of myself	0	1	2	3	A
10	I felt that I had nothing to look forward to	0	1	2	3	D
11	I found myself getting agitated	0	1	2	3	S

12	I found it difficult to relax	0	1	2	3	S
13	I felt down-hearted and blue	0	1	2	3	D
14	I was intolerant of anything that kept me from getting on with what I was doing	0	1	2	3	S
15	I felt I was close to panic	0	1	2	3	A
16	I was unable to become enthusiastic about anything	0	1	2	3	D
17	I felt I was not worth much as a person	0	1	2	3	D
18	I felt that I was rather touchy	0	1	2	3	S
19	I was aware of the action of my heart in the absence of physical exertion (e.g., sense of heart rate increase, heart missing a beat)	0	1	2	3	A
20	I felt scared without any good reason	0	1	2	3	A
21	I felt that life was meaningless	0	1	2	3	D

Severity ratings of DASS scale

Severity	Depression	Anxiety	Stress
Normal	0–9	0–7	0–14
Mild	10–13	8–9	15–18
Moderate	14–20	10–14	19–25
Severe	21–27	15–19	26–33
Extremely severe	>27	>19	>33
