# Supplementary material for: Does psychological distress influence postoperative satisfaction and outcomes in patients undergoing total knee arthroplasty? A prospective cohort study
Source: BMC Musculoskelet Disord. 2021 Jul 30;22:647. doi: 10.1186/s12891-021-04528-7 (PMC8325222; doi:10.1186/s12891-021-04528-7)
Supplement: Supplementary file 2 — Additional file 2: Online Resource 2. Knee Society Scores at different time points among patients with different numbers of distress characteristics. [file 12891_2021_4528_MOESM2_ESM.pdf]

Article title: Does psychological distress influence postoperative satisfaction and outcomes in patients undergoing total knee arthroplasty? A prospective cohort study

Journal name: BMC Musculoskeletal Disorders

Author names: Tao Bian, Hongyi Shao, Yixin Zhou, Yong Huang, Yang Song

Corresponding Author: Yixin Zhou
Department of Orthopedic Surgery, Beijing Jishuitan Hospital, Fourth Clinical College of Peking University, No. 31 Xinjiekou East Street, Xicheng District, Beijing 100035, China
E-mail: orthoyixin@yahoo.com

Online Resource 2. Knee Society Scores at different time points among patients with different numbers of distress characteristics

Outcome measure, median (IQR)		Pre-operatively	3 Months	1 Year	Difference in Knee Society Scores
Median Knee Society function score (IQR)	Three distress characteristics	32.0 (19.0 to 49.3)	43.5 (34.0 to 50.5)	54.0 (43.3 to 60.3)	21.0 (7.5 to 32.3)
	Two distress characteristics	29.0 (19.0 to 38.3)	43.0 (35.8 to 49.3)	57.0 (45.5 to 62.3)	28.0 (9.5 to 37.0)

	One distress characteristic	30.0 (19.0 to 44.5)	40.0 (30.0 to 48.5)	55.0 (46.0 to 60.5)	24.0 (11.0 to 32.5)
	No distress	38.0 (26.0 to 52.0)	40.0 (34.5 to 50.5)	54.0 (47.0 to 60.0)	16.0 (-0.5 to 29.0)
	P value	0.026*	0.558	0.775	0.035*
Median Knee Society symptom score (IQR)	Three distress characteristics	7.0 (3.8 to 8.5)	21.0 (16.0 to 23.5)	23.0 (21.0 to 25.0)	15.0 (12.8 to 21.0)
	Two distress characteristics	6.0 (3.0 to 8.0)	19.0 (14.0 to 23.5)	25.0 (22.0 to 25.0)	17.5 (13.0 to 20.3)
	One distress characteristic	8.0 (3.5 to 12.0)	19.0 (17.0 to 24.5)	25.0 (23.0 to 25.0)	15.0 (12.0 to 21.0)
	No distress	8.0 (5.0 to 13.0)	21.0 (17.0 to 23.0)	25.0 (23.0 to 25.0)	15.0 (11.0 to 19.0)
	P value	0.096	0.751	0.314	0.282

Median Knee Society objective score (IQR)	Three distress characteristics	26.5 (11.8 to 41.3)	63.5 (54.5 to 66.0)	64.0 (34.3 to 67.0)	23.0 (6.8 to 50.5)
	Two distress characteristics	25.5 (7.0 to 34.0)	65.0 (63.0 to 67.0)	64.0 (61.3 to 68.3)	37.0 (23.8 to 54.5)
	One distress characteristic	25.0 (11.5 to 35.0)	65.0 (63.0 to 69.0)	65.0 (63.5 to 68.0)	39.0 (24.0 to 51.5)
	No distress	29.0 (11.0 to 43.5)	65.0 (61.0 to 67.0)	65.0 (62.5 to 68.0)	31.0 (8.5 to 46.5)
	P value	0.501	0.233	0.349	0.160
Median Knee Society	Three distress characteristics	15.0 (13.8 to 15.0)	9.0 (6.0 to 9.0)	9.0 (7.5 to 9.0)	-6.0 (-6.25 to -4.8)

expectation score (IQR)	Two distress characteristics	14.5 (12.0 to 15.0)	9.0 (6.0 to 9.0)	9.0 (9.0 to 9.0)	-6.0 (-6.0 to -2.8)
	One distress characteristic	15.0 (13.5 to 15.0)	9.0 (6.0 to 9.0)	9.0 (8.5 to 9.0)	-6.0 (-6.0 to -4.5)
	No distress	14.0 (12.0 to 15.0)	9.0 (7.0 to 9.0)	9.0 (8.5 to 9.0)	-6.0 (-6.0 to -4.0)
	P value	0.168	0.990	0.590	0.455
Median Knee Society satisfaction score (IQR)	Three distress characteristics	10.0 (7.0 to 18.5)	24.0 (20.0 to 30.0)	30.0 (20.0 to 30.0)	18.0 (7.0 to 20.5)
	Two distress characteristics	10.0 (6.0 to 16.0)	24.0 (20.0 to 30.0)	30.0 (25.5 to 30.0)	14.0 (10.0 to 22.5)
	One distress characteristic	12.0 (8.0 to 18.0)	30.0 (22.0 to 30.0)	30.0 (27.0 to 30.0)	16.0 (10.0 to 20.0)

No distress	14.0 (10.0 to 20.0)	30.0 (22.0 to 30.0)	30.0 (25.0 to 30.0)	12.0 (8.0 to 20.0)
P value	0.063	0.450	0.460	0.218

Abbreviation: IQR, interquartile range. *There was no significant difference in pairwise comparisons.
