# Supplementary material for: Does psychological distress influence postoperative satisfaction and outcomes in patients undergoing total knee arthroplasty? A prospective cohort study
Source: BMC Musculoskelet Disord. 2021 Jul 30;22:647. doi: 10.1186/s12891-021-04528-7 (PMC8325222; doi:10.1186/s12891-021-04528-7)
Supplement: Supplementary file 3 — Additional file 3: Online Resource 3. Knee Society Scores at different time points among patients with different severity scores of depression. [file 12891_2021_4528_MOESM3_ESM.pdf]

Article title: Does psychological distress influence postoperative satisfaction and outcomes in patients undergoing total knee arthroplasty? A prospective cohort study

Journal name: BMC Musculoskeletal Disorders

Author names: Tao Bian, Hongyi Shao, Yixin Zhou, Yong Huang, Yang Song

Corresponding Author: Yixin Zhou
Department of Orthopedic Surgery, Beijing Jishuitan Hospital, Fourth Clinical College of Peking University, No. 31 Xinjiekou East Street, Xicheng District, Beijing 100035, China
E-mail: orthoyixin@yahoo.com

Online Resource 3. Knee Society Scores at different time points among patients with different severity scores of depression

Outcome measure, median (IQR)		Pre-operatively	3 Months	1 Year	Difference in Knee Society Scores
Median Knee Society function score (IQR)	Severe depression	24.0 (11.0 to /)	44.0 (14.0 to /)	47.0 (16.0 to /)	22.0 (5.0 to /)
	Moderate depression	32.0 (18.0 to 45.0)	43.0 (33.0 to 48.5)	58.0 (54.0 to 63.5)	23.0 (9.5 to 33.5)
	Mild depression	32.0 (19.0 to 48.0)	41.0 (35.0 to 48.0)	53.0 (41.0 to 64.0)	19.0 (-3.0 to 34.0)

	No depression	35.0 (24.0 to 48.0)	41.0 (34.0 to 50.0)	54.0 (47.0 to 60.0)	19.0 (2.0 to 30.0)
	P value	0.243	0.937	0.185	0.677
Median Knee Society symptom score (IQR)	Severe depression	0.0	19.0 (19.0 to /)	21.0 (16.0 to /)	21.0 (7.0 to /)
	Moderate depression	6.0 (4.0 to 7.5)	19.0 (11.0 to 24.0)	25.0 (21.0 to 25.0)	17.0 (14.5 to 19.5)
	Mild depression	8.0 (3.0 to 12.0)	19.0 (14.0 to 25.0)	24.0 (21.0 to 25.0)	14.0 (10.0 to 21.0)
	No depression	8.0 (5.0 to 12.0)	21.0 (17.0 to 23.0)	25.0 (23.0 to 25.0)	16.0 (12.0 to 20.0)
	P value	0.114	0.768	0.066	0.756
	Severe depression	15.0 (5.0 to /)	66.0 (65.0 to /)	67.0 (65.0 to /)	55.0 (49.0 to /)

Median Knee Society objective score (IQR)	Moderate depression	24.0 (0.0 to 40.0)	64.0 (58.5 to 66.0)	64.0 (58.5 to 68.0)	34.0 (17.5 to 67.5)
	Mild depression	28.0 (12.0 to 39.0)	65.0 (57.0 to 67.0)	64.0 (35.0 to 67.0)	34.0 (15.0 to 45.0)
	No depression	28.0 (12.0 to 40.0)	65.0 (62.0 to 67.0)	65.0 (63.0 to 68.0)	35.0 (14.0 to 48.0)
	P value	0.265	0.428	0.183	0.165
Median Knee Society expectation score (IQR)	Severe depression	15.0 (15.0 to 15.0)	9.0 (6.0 to /)	8.0 (3.0 to /)	-7.0 (-12.0 to /)
	Moderate depression	15.0 (14.0 to 15.0)	9.0 (6.0 to 9.0)	9.0 (9.0 to 9.0)	-6.0 (-6.0 to -5.0)
	Mild depression	15.0 (12.0 to 15.0)	9.0 (8.0 to 9.0)	9.0 (6.0 to 9.0)	-6.0 (-6.0 to -3.0)
	No depression	15.0 (13.0 to 15.0)	9.0 (7.0 to 9.0)	9.0 (9.0 to 9.0)	-6.0 (-6.0 to -4.0)
	P value	0.358	0.982	0.262	0.138

Median Knee Society satisfaction score (IQR)	Severe depression	0.0	24.0 (20.0 to /)	26.0 (10.0 to /)	26.0 (2.0 to /)
	Moderate depression	10.0 (5.0 to 14.0)	24.0 (20.0 to 30.0)	30.0 (30.0 to 30.0)	20.0 (16.0 to 24.0)
	Mild depression	10.0 (8.0 to 18.0)	24.0 (20.0 to 30.0)	30.0 (20.0 to 30.0)	14.0 (8.0 to 20.0)
	No depression	14.0 (10.0 to 20.0)	30.0 (22.0 to 30.0)	30.0 (26.0 to 30.0)	14.0 (8.0 to 20.0)
	P value	0.011*	0.130	0.078	0.214

Abbreviation: IQR, interquartile range. *There was no significant difference in pairwise comparisons.
