# Supplementary material for: Does psychological distress influence postoperative satisfaction and outcomes in patients undergoing total knee arthroplasty? A prospective cohort study
Source: BMC Musculoskelet Disord. 2021 Jul 30;22:647. doi: 10.1186/s12891-021-04528-7 (PMC8325222; doi:10.1186/s12891-021-04528-7)
Supplement: Supplementary file 4 — Additional file 4: Online Resource 4. Knee Society Scores at different time points among patients with different severity scores of anxiety. [file 12891_2021_4528_MOESM4_ESM.pdf]

Article title: Does psychological distress influence postoperative satisfaction and outcomes in patients undergoing total knee arthroplasty? A prospective cohort study

Journal name: BMC Musculoskeletal Disorders

Author names: Tao Bian, Hongyi Shao, Yixin Zhou, Yong Huang, Yang Song

Corresponding Author: Yixin Zhou
Department of Orthopedic Surgery, Beijing Jishuitan Hospital, Fourth Clinical College of Peking University, No. 31 Xinjiekou East Street, Xicheng District, Beijing 100035, China
E-mail: orthoyixin@yahoo.com

Online Resource 4. Knee Society Scores at different time points among patients with different severity scores of anxiety

Outcome measure, median (IQR)		Pre-operatively	3 Months	1 Year	Difference in Knee Society Scores
Median Knee Society function score (IQR)	Extremely severe anxiety	24.5 (19.8 to 45.3)	44.0 (42.5 to 52.0)	52.5 (43.8 to 57.5)	20.5 (5.8 to 40.0)
	Severe anxiety	32.0 (25.0 to 41.0)	48.0 (35.0 to 53.0)	59.0 (54.0 to 64.0)	23.0 (18.0 to 34.0)
	Moderate anxiety	32.0 (18.0 to 45.0)	41.0 (29.5 to 47.5)	55.0 (44.0 to 62.5)	22.0 (12.5 to 32.0)

	Mild anxiety	29.0 (24.0 to 42.3)	36.0 (30.3 to 43.8)	53.0 (46.5 to 57.5)	27.0 (-0.8 to 34.8)
	No anxiety	36.5 (26.0 to 50.8)	40.5 (34.3 to 50.8)	54.0 (47.0 to 60.0)	16.0 (0.0 to 29.8)
	P value	0.172	0.166	0.248	0.129
Median Knee Society symptom score (IQR)	Extremely severe anxiety	3.5 (0.0 to 7.8)	23.0 (19.0 to 25.0)	24.5 (22.5 to 25.0)	21.0 (16.5 to 23.0)
	Severe anxiety	7.0 (4.0 to 9.0)	21.0 (17.0 to 25.0)	23.0 (22.0 to 25.0)	17.0 (13.0 to 21.0)
	Moderate anxiety	6.0 (4.0 to 10.5)	19.0 (15.5 to 24.0)	25.0 (22.0 to 25.0)	17.0 (12.0 to 20.0)
	Mild anxiety	9.0 (3.8 to 12.0)	19.0 (15.5 to 22.0)	25.0 (23.0 to 25.0)	15.0 (11.5 to 20.8)
	No anxiety	8.0 (5.0 to 12.8)	21.0 (16.3 to 23.0)	25.0 (23.0 to 25.0)	15.0 (11.0 to 18.8)
	P value	0.067	0.496	0.714	0.149

Median Knee Society objective score (IQR)	Extremely severe anxiety	23.5 (11.3 to 39.3)	65.0 (64.0 to 70.0)	64.5 (58.5 to 68.3)	36.5 (22.5 to 58.3)
	Severe anxiety	21.0 (8.0 to 37.0)	65.0 (59.0 to 67.0)	64.0 (32.0 to 65.0)	22.0 (14.0 to 57.0)
	Moderate anxiety	28.0 (15.5 to 40.0)	65.0 (57.0 to 68.0)	65.0 (63.0 to 67.0)	35.0 (15.5 to 45.0)
	Mild anxiety	23.0 (8.0 to 33.5)	65.0 (63.0 to 68.8)	65.0 (63.3 to 69.0)	40.0 (29.3 to 52.3)
	No anxiety	28.5 (10.3 to 42.3)	65.0 (61.0 to 67.0)	65.0 (63.0 to 68.8)	32.5 (9.3 to 49.8)
	P value	0.624	0.523	0.438	0.576
Median Knee Society expectation score (IQR)	Extremely severe anxiety	15.0 (14.0 to 15.0)	9.0 (8.3 to 9.8)	9.0 (8.8 to 9.8)	-6.0 (-6.3 to -3.0)
	Severe anxiety	15.0 (15.0 to 15.0)	9.0 (9.0 to 9.0)	9.0 (9.0 to 9.0)	-6.0 (-6.0 to -6.0)
	Moderate anxiety	15.0 (12.0 to 15.0)	9.0 (6.0 to 9.0)	9.0 (8.0 to 9.0)	-6.0 (-6.0 to -3.5)
	Mild anxiety	15.0 (13.0 to 15.0)	9.0 (6.0 to 9.0)	9.0 (9.0 to 9.0)	-6.0 (-6.0 to -4.3)

	No anxiety	14.0 (12.3 to 15.0)	9.0 (7.0 to 9.0)	9.0 (8.3 to 9.0)	-6.0 (-6.0 to -4.0)
	P value	0.254	0.713	0.838	0.835
Median Knee Society satisfaction score (IQR)	Extremely severe anxiety	19.0 (7.5 to 22.5)	24.0 (23.5 to 30.0)	30.0 (29.0 to 30.0)	11.0 (7.5 to 21.5)
	Severe anxiety	10.0 (6.0 to 12.0)	30.0 (22.0 to 30.0)	30.0 (24.0 to 24.0)	20.0 (12.0 to 22.0)
	Moderate anxiety	12.0 (7.0 to 18.0)	30.0 (20.0 to 30.0)	30.0 (24.0 to 30.0)	16.0 (9.0 to 20.0)
	Mild anxiety	14.0 (10.5 to 16.0)	29.0 (20.0 to 30.0)	30.0 (25.5 to 30.0)	14.0 (10.0 to 17.5)
	No anxiety	14.0 (10.0 to 20.0)	30.0 (22.0 to 30.0)	30.0 (24.5 to 30.0)	13.0 (8.0 to 20.0)
	P value	0.040*	0.908	0.865	0.164

Abbreviation: IQR, interquartile range. *There was no significant difference in pairwise comparisons.
