# Supplementary material for: Does psychological distress influence postoperative satisfaction and outcomes in patients undergoing total knee arthroplasty? A prospective cohort study
Source: BMC Musculoskelet Disord. 2021 Jul 30;22:647. doi: 10.1186/s12891-021-04528-7 (PMC8325222; doi:10.1186/s12891-021-04528-7)
Supplement: Supplementary file 5 — Additional file 5: Online Resource 5. Knee Society Scores at different time points among patients with different severity scores of stress. [file 12891_2021_4528_MOESM5_ESM.pdf]

Article title: Does psychological distress influence postoperative satisfaction and outcomes in patients undergoing total knee arthroplasty? A prospective cohort study

Journal name: BMC Musculoskeletal Disorders

Author names: Tao Bian, Hongyi Shao, Yixin Zhou, Yong Huang, Yang Song

Corresponding Author: Yixin Zhou
Department of Orthopedic Surgery, Beijing Jishuitan Hospital, Fourth Clinical College of Peking University, No. 31 Xinjiekou East Street, Xicheng District, Beijing 100035, China
E-mail: orthoyixin@yahoo.com

Online Resource 5. Knee Society Scores at different time points among patients with different severity scores of stress

Outcome measure, median (IQR)		Pre-operatively	3 Months	1 Year	Difference in Knee Society Scores
Median Knee Society function score (IQR)	Severe stress	30.5 (24.3 to 46.5)	45.0 (41.8 to 55.0)	52.0 (44.8 to 63.0)	25.5 (1.0 to 32.0)
	Moderate stress	32.0 (28.3 to 40.8)	48.0 (36.8 to 61.8)	55.5 (51.3 to 64.8)	23.0 (16.3 to 33.0)
	Mild stress	26.5 (18.3 to 40.5)	44.0 (35.0 to 48.0)	54.0 (41.5 to 59.8)	23.0 (13.3 to 33.0)

	No stress	35.0 (23.3 to 48.0)	41.0 (34.0 to 50.0)	55.0 (46.3 to 60.0)	18.0 (1.3 to 32.0)
	P value	0.603	0.317	0.804	0.622
Median Knee Society symptom score (IQR)	Severe stress	1.5 (0.0 to 5.3)	21.0 (19.0 to 23.0)	24.0 (21.5 to 25.0)	21.5 (19.5 to 22.8)
	Moderate stress	6.0 (3.0 to 10.5)	25.0 (12.5 to 25.0)	25.0 (22.5 to 25.0)	16.5 (14.5 to 21.3)
	Mild stress	7.5 (5.3 to 8.0)	19.0 (13.3 to 23.0)	24.0 (21.0 to 25.0)	17.0 (12.3 to 18.5)
	No stress	8.0 (4.3 to 12.0)	20.0 (17.0 to 23.0)	25.0 (23.0 to 25.0)	15.0 (11.3 to 20.0)
	P value	0.088	0.459	0.717	0.117
Median Knee Society objective score (IQR)	Severe stress	39.5 (15.3 to 70.5)	64.5 (58.8 to 68.8)	67.5 (64.3 to 70.0)	28.0 (-5.0 to 53.5)
	Moderate stress	26.5 (20.0 to 37.5)	61.5 (48.3 to 66.3)	47.5 (31.5 to 64.5)	13.0 (3.8 to 40.8)

	Mild stress	30.0 (2.0 to 40.0)	64.5 (55.8 to 66.5)	65.0 (58.5 to 67.0)	32.0 (16.3 to 56.8)
	No stress	27.0 (11.3 to 40.0)	65.0 (63.0 to 67.0)	65.0 (63.0 to 68.0)	36.0 (18.3 to 48.8)
	P value	0.839	0.676	0.116	0.589
Median Knee Society expectation score (IQR)	Severe stress	15.0 (15.0 to 15.0)	9.0 (6.8 to 9.0)	8.5 (6.5 to 9.0)	-6.5 (-8.5 to -6.0)
	Moderate stress	15.0 (14.3 to 15.0)	9.0 (7.5 to 9.8)	9.0 (9.0 to 9.8)	-6.0 (-6.0 to -3.0)
	Mild stress	14.5 (13.3 to 15.0)	9.0 (6.0 to 9.0)	9.0 (9.0 to 9.0)	-6.0 (-6.0 to -4.3)
	No stress	15.0 (12.3 to 15.0)	9.0 (7.0 to 9.0)	9.0 (9.0 to 9.0)	-6.0 (-6.0 to -4.0)
	P value	0.246	0.697	0.308	0.117
	Severe stress	9.0 (0.0 to 22.5)	24.0 (21.0 to 28.5)	28.0 (21.5 to 30.0)	19.0 (0.0 to 29.0)

Median Knee Society satisfaction score (IQR)	Moderate stress	10.0 (6.5 to 11.0)	30.0 (19.5 to 30.0)	30.0 (30.0 to 30.0)	20.0 (19.0 to 23.5)
	Mild stress	11.0 (8.0 to 19.0)	24.0 (20.0 to 30.0)	30.0 (21.0 to 30.0)	15.0 (8.0 to 21.5)
	No stress	14.0 (10.0 to 20.0)	30.0 (22.0 to 30.0)	30.0 (24.5 to 30.0)	14.0 (8.0 to 20.0)
	P value	0.174	0.886	0.365	0.268

Abbreviation: IQR, interquartile range.
